# Supplementary figures and images for: Chasing high and stable wheat grain mineral content: Mining diverse spring genotypes under induced drought stress
Source: PLoS One. 2024 Feb 15;19(2):e0298350. doi: 10.1371/journal.pone.0298350 (PMC10868752; doi:10.1371/journal.pone.0298350)

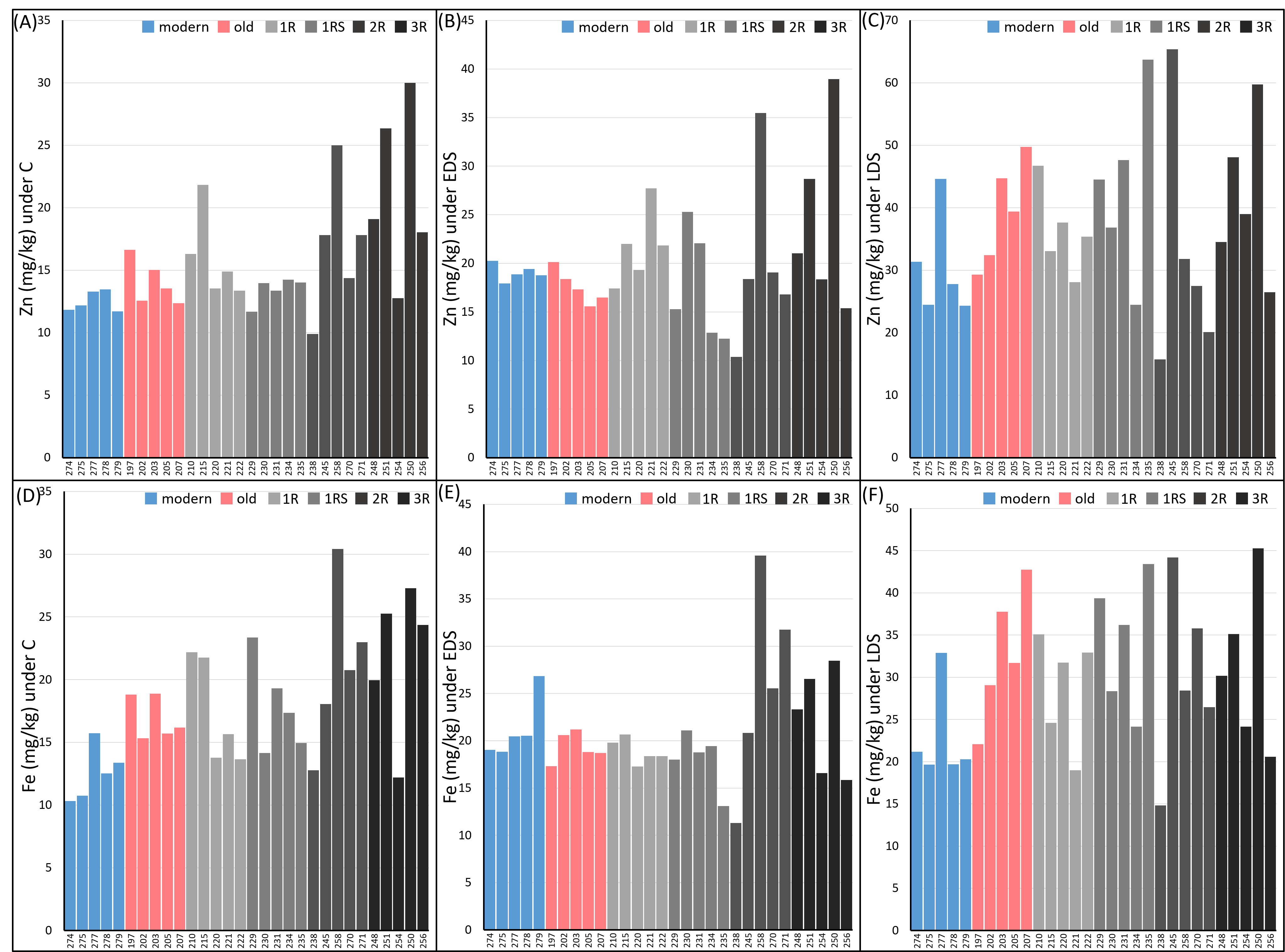

Supplement: S1 Fig — Zn (A-C) and Fe (D-E) concentration of each genotype under control (C), early drought stress (EDS) and late drought stress (LDS). Modern = approved cultivars and breeding lines received from company Lantmännen; old = old Swedish cultivars released from 1928 to 1990; 1R, 1RS, 2R and 3R = Introgressions of chromosome 1R, 1RS, 2R and 3R. The value of each genotype was generated from the mean of three biological replicates. (TIF) [file pone.0298350.s001.tif]

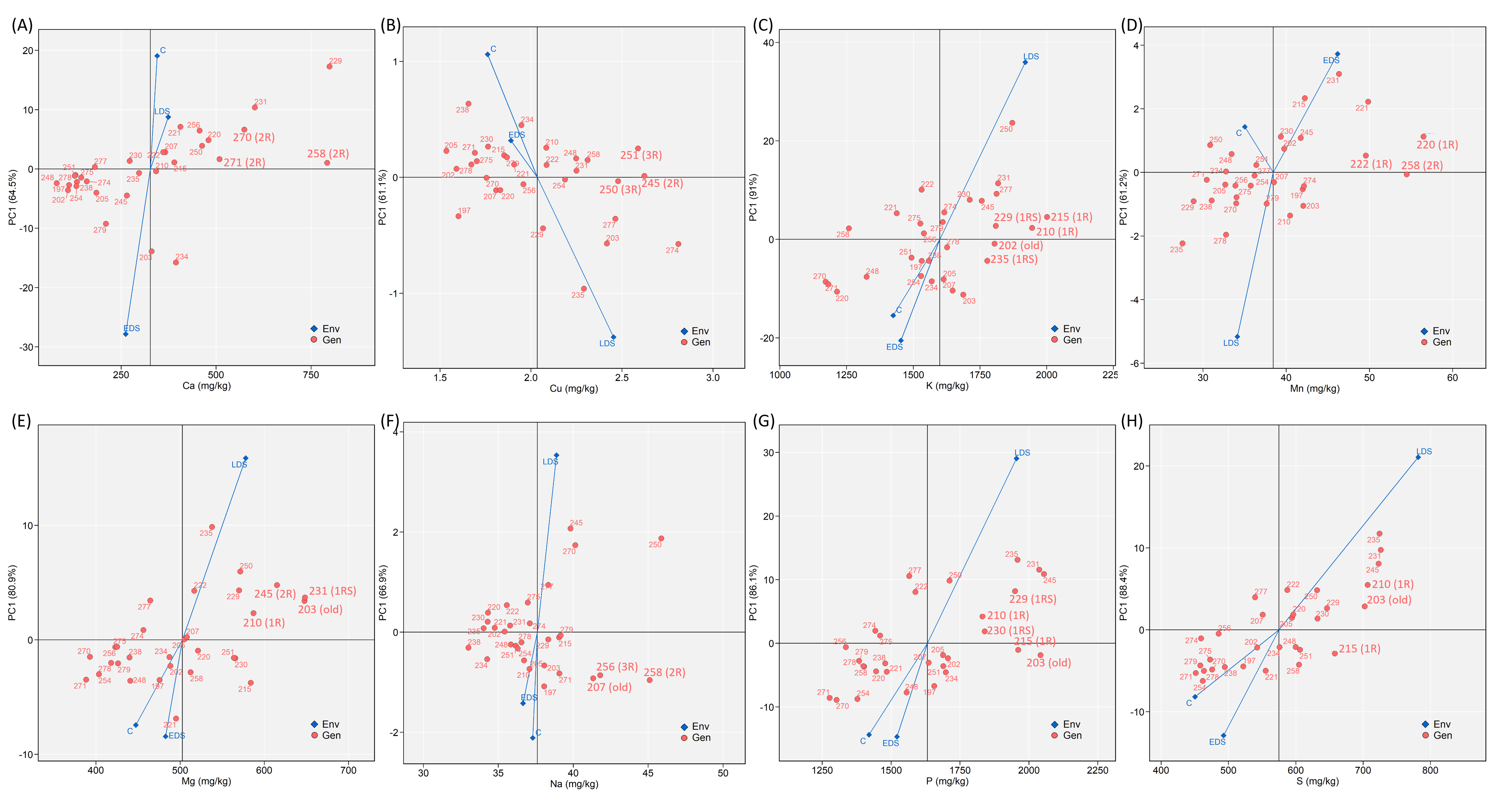

Supplement: S2 Fig — Additive main effects and multiplicative interaction (AMMI) biplots showing concentration of (A) Ca, (B) Cu, (C) K, (D) Mn, (E) Mg, (F) Na, (G) P and (H) S versus the first principal component (PC1) score of 30 genotypes (Gen) and three growing conditions (Env) including control (abbreviated as C), early drought stress (EDS) and late drought stress (LDS). Genotypes located closer to the horizontal axis (score 0 on PC1) are those showing relatively higher stability across the three growing conditions. The vertical line in each figure indicates the average mineral concentration of the 30 genotypes. (TIF) [file pone.0298350.s002.tif]

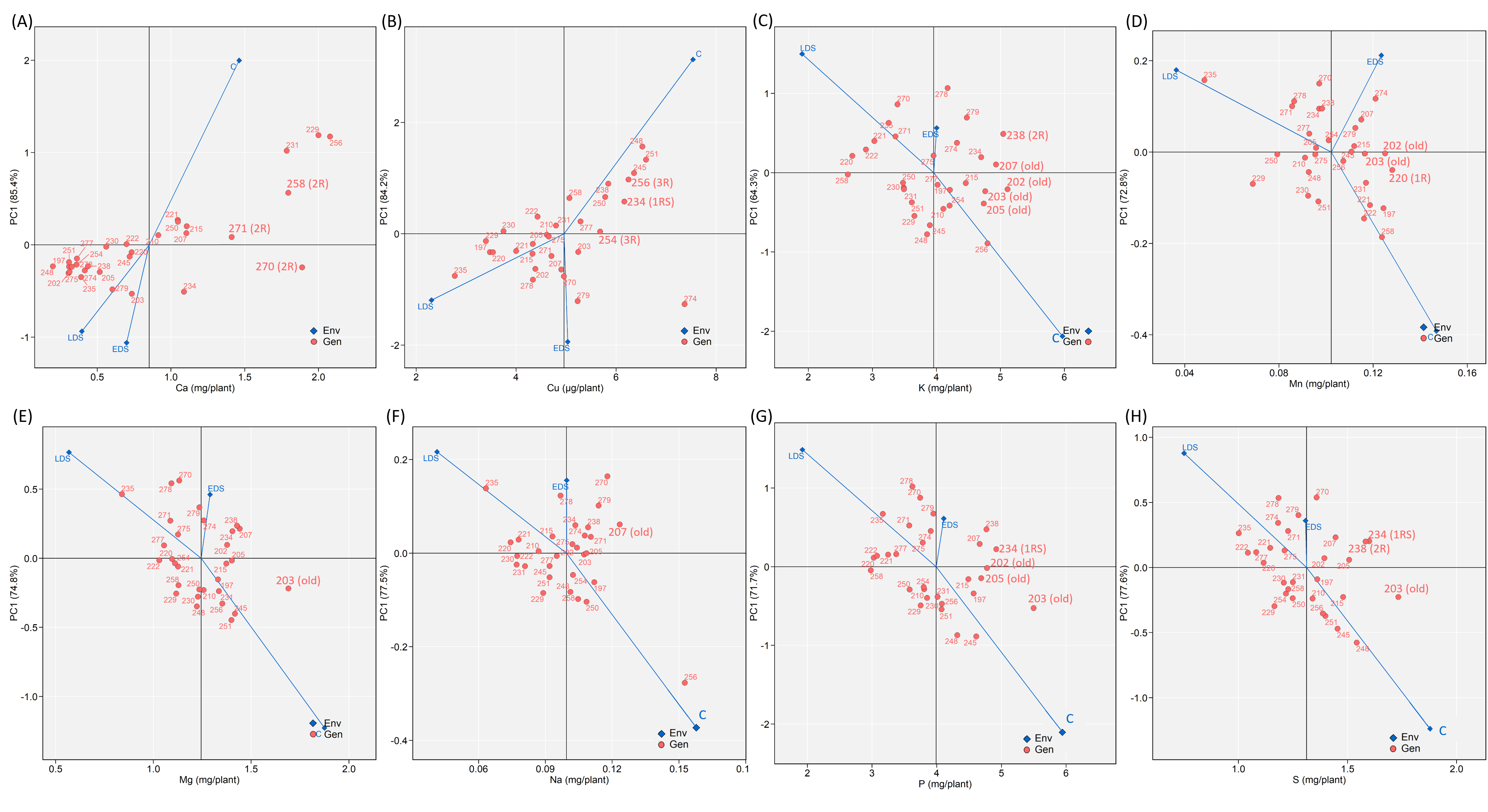

Supplement: S3 Fig — Additive main effects and multiplicative interaction (AMMI) biplots showing mineral yield of (A) Ca, (B) Cu, (C) K, (D) Mn, (E) Mg, (F) Na, (G) P and (H) S versus the first principal component (PC1) score of 30 genotypes (Gen) and three growing conditions (Env) including control (abbreviated as C), early drought stress (EDS) and late drought stress (LDS). Genotypes located closer to the horizontal axis (score 0 on PC1) are those showing relatively higher stability across the three growing conditions. The vertical line in each figure indicates the average mineral yield of the 30 genotypes. (TIF) [file pone.0298350.s003.tif]
