# Supplementary material for: Chasing high and stable wheat grain mineral content: Mining diverse spring genotypes under induced drought stress
Source: PLoS One. 2024 Feb 15;19(2):e0298350. doi: 10.1371/journal.pone.0298350 (PMC10868752; doi:10.1371/journal.pone.0298350)
Supplement: Attachment — Submitted filename: Response to Reviewers.docx [file pone.0298350.s007.docx]

**Point-by-point answers to Reviewers comments**

Dear Editor,

We appreciate the valuable comments and suggestions from the reviewers, which we have tried to constructively use to improve the quality of our manuscript. The manuscript has now been revised following every point of the reviewers suggestions. Changes in the manuscript were made according to reviewers’ comments and are addressed point by point as below:

Reviewer 1.

The work is extremely interesting and valuable but to my opinion there are some drawbacks.

Thanks for your positive comment about the value of our work.

Major comments

Material and Methods section is extremely short and should be seriously revised. The authors suggest the readers to read their previous article published in another journal!!!!~ Such a proposal may be OK only in case the previous work has been published in the same journal. Please, add full description of the experiment, soil characteristics, Se, Fe and Zn content in soil, the dates of seeds sowing and harvest, the number of genotypes in each group studied, drought treatment, etc.

Thanks for the suggestion. A detailed description of the experiment has now been added in Material and Methods (Line 86-88, 90-92, 94-103), providing data as per suggestion from the reviewer. As for the soil characteristics, we have added all information about the soil that was provided by the producer. Unfortunately, the content of specific Se, Fe and Zn in the soil was not given by the provider, but we have added all the available information about the soil characteristics including soil component information, pH, product name, article number and provider name for the readers to reproduce the experiment. All the plants were grown in the same soil product produced by the same company, which gives different genotypes a uniform chemical condition. Therefore, we are sure that the differences of mineral concentrations found between genotype groups are valid and the comparison made between different genotypes can represent the genetic difference of those attributes. If the editor so prefer, we can analyse the content of the mentioned specific compounds in the soil, although not of the same batch used in the experiment as this batch is used up. However, as the soil comes from natural peat sources, there might be a slight risk of variation in the mentioned compounds between batches, which makes such measures less useful.

1) Use Italics for the Latin name of wheat.

Thanks for the correction. Italics has been applied to the Latin name of wheat (Line 91).

2) The manuscript is overloaded with abbreviations which hinders the perception of the data. Please, don’t abbreviate at least ‘control’ in the text and change ‘C’ to ‘control’. Abbreviations are useful in Figures but extremely tedious in the text.

Thanks for the suggestion. We agree that using ‘C’ in the main texts can be confusing. Therefore, all the ‘C’s indicating the control condition have now been changed into ‘control’ in the main texts.

3) Line 102 mineral determination: what was the internal and external reference standards for the elements tested?

Thanks for the comment. Additional information as related to the mineral determinations have been added to the manuscript (Line 126-131).

4) Line 42 I can’t agree with the statement that ‘sulfur (S) is mainly supplied to humans in the form of methionine’. What about cysteine, sulfur derivatives in Allium species, glucosinolates in Brassica plants???

Thank you for the comments. The description has been corrected as suggested. Different dietary Sulfur sources have been clarified (Line 48-52).

5) The title does not provide a concrete information about the content of the work. May be, it is better to change it to ‘The impacts of early and late drought stress on wheat grain mineral composition”?

Thanks for the suggestion. To provide more concrete information about our work, we have modified the title.

6) It is highly desirable to add grain yield for different genotypes and genotype groups

The data for grain yield have been added along with other mineral data as a supporting file (S2 Table).

7) Is there any information about the differences in grain quality except minerals?

Yes, the genotypes used here have also been subjected to grain quality analyses along with a larger set of wheat lines but the grain quality data have been published in another paper. We have added further discussions on the grain-quality differences among the studied genotypes, especially the 3R versus other introgression genotypes, and modern wheat versus all the introgression genotypes (Line 437-439, 489-493).

8) While speaking about the relationship between the elements (line 203, Zn, Fe) the significance of the correlation should be indicated. Please, add ‘p<’

Thanks for pointing out the missing P values to indicate the significance. It is now added to each plot as recommended.

9) line 425 ‘The uptake mechanisms of Se are related to the two major chemical forms present in soil, i.e. selenate and selenite, as these are transferred by sulfate and phosphate transporters, respectively’- what about organic Se and Se-S interaction?

Thanks for the comments. As suggested, further discussions on Se-S interaction and the organic form of Se in soils have been added in the discussion section (Line 459-464) accompanied by some necessary citations.

10) The authors have forgotten about the work of Ayed, S.; Bouhaouel, I.; Othmani, A.: Screening of Durum Wheat Cultivars for Selenium Response under Contrasting Environments, Based on Grain Yield and Quality Attributes. Plants 2022, 11, 1437. https://doi.org/10.3390/ plants11111437. Please add this citation in Introduction/Discussion sections and in the reference list

Thanks for the suggestion. The recommended citation has been added in the discussion section (Line 443-444) with some texts to describe the effect of Se on wheat.

11) Line 490: a misprint: ‘aluminium’ change to ‘aluminum’.

Thanks for pointing out this. As far as we know both ‘aluminium’ and ‘aluminum’ are correct. However, there is one paper listed in the References with a title using ‘aluminum’. To unify the term used throughout the whole manuscript, the ‘aluminium’ pointed out by the reviewer has been replaced with ‘aluminum’ as recommended.

Reviewer 2.

The manuscript is technically sound, and the presented data support the conclusions. Appropriate and rigorous statistical analysis is performed. Moreover, the manuscript is presented in an intelligible fashion and written in standard English. Thus, I confidently recommend to accept the manuscript for publication in POLS ONE.

Thanks for the positive comments about our manuscript.

As can be seen from the point-by-point answers to reviewer comments, we have made every attempt to improve the manuscript following suggestions from the reviewers. We thereby hope that the manuscript is now suitable for publication in PLOS ONE.
